# Complete chloroplast genome of *Kadsura coccinea* (Lem.) A.C.Sm. (Schisandraceae): genome structure and evolution

**DOI:** 10.1080/23802359.2021.1904798

**Published:** 2021-03-30

**Authors:** Hui-Zhen Qin, Li-Li Deng, Yan-Cai Shi

**Affiliations:** Guangxi Institute of Botany, Guangxi Zhuang Autonomous Region and Chinese Academy of Sciences, Guilin, China

**Keywords:** Chloroplast genome, phylogenetic analysis, Schisandraceae, *Kadsura coccinea*

## Abstract

*Kadsura coccinea* (Lem.) A.C.Sm. in the Schisandraceae family is woody vine plant, which produce edible red fruits that are rich in nutrients and antioxidant activities. Herein, we assembled the complete chloroplast genome of *Kadsura coccinea* by next-generation sequencing technologies. The complete chloroplast genome sequence of *Kadsura coccinea* is 145,413 base pairs (bp) in length, including a pair of inverted repeat regions (IRs, 16,431 bp), one large single-copy region (LSC, 94,511 bp), one small single-copy region (SSC, 18,040 bp). Besides, the complete chloroplast genome contains 126 genes in total, including 82 protein-coding genes, 35 tRNA genes, and 8 rRNA genes. Phylogenetic analysis showed that *Kadsura coccinea* has the closest relationship with *Kadsura longipedunculata*. Our study lay a foundation for further research of *Kadsura coccinea*.

*Kadsura coccinea* is a kind of new type of wild fruit for both medicine and food. It is rich in lignans, amino acids, anthocyanins and other trace elements (Xie et al. 2016). It has the functions of anti-hepatitis, anti-oxidation and neuroprotection (Xie 2019). The genetic diversity makes its progeny segregation seriously, and the yield is low. Despite their valuable food applications, *Kadsura coccinea* is only able to grow naturally in the forest, and reproduction handled by botanists is still in progress with a very low growth rate. Subsequently, *Kadsura coccinea* was listed as endangered species by the International Union for Conservation of Nature and Natural Resources (IUCN) in 2011 (Sritalahareuthai et al. 2020). However, there are very few studies on the *Kadsura coccinea*, which greatly limit the development and utilization of *Kadsura coccinea*. In this study, we assembled the complete chloroplast genome of *Kadsura coccinea*, hoping to lay a foundation for further research.

The samples of *Kadsura coccinea* was collected from Yachang Orchidaceae National Nature Reserve, Guangxi, China (24°44'N, 106°15'E) and the voucher specimen deposited at Herbarium of Guangxi Institute of Botany, Guangxi Zhuang Autonomous Region and Chinese Academy of Sciences (specimen code *Coccinea*_GX).

High-quality genomic DNA of *Kadsura coccinea* was extracted from leaves by TIANGEN plant genomic DNA kit, and sequenced by the BGISEQ-500 platform. With the chloroplast genome of *Kadsura longipedunculata* (GenBank accession number MH_535482) as the reference sequences, we assembled the complete chloroplast genome from the clean reads by the GetOrganelle pipe-line (Jin et al. 2018), and then annotated the new sequences using the Geneious R11.15 (Kearse et al. 2012). Finally, a complete chloroplast genome of *Kadsura coccinea* was obtained and submitted to Genbank (accession number MT934443) and BioSample metadata are available in the NCBI BioSample database (http://www.ncbi.nlm.nih.gov/biosample/) under accession number SRR13766590.

The complete chloroplast genome sequence of *Kadsura coccinea* is 145,413 base pairs (bp) in length, including a pair of inverted repeat regions (IRs, 16,431 bp), one large single-copy region (LSC, 94,511 bp), one small single-copy region (SSC, 18,040 bp). Besides, the complete chloroplast genome contains 126 genes in total, including 82 protein-coding genes, 35 tRNA genes, and 8 rRNA genes. In addition, the overall GC content of the genome was 39.7%. Subtle differences from other researchers, the chloroplast genome of *Kadsura coccinea* (MH029822) with two inverted repeats (each 16,536 bp in length) separated by one large single-copy region and one small single-copy region (94,301 and 18,040 bp in length, respectively). The chloroplast genome of *Kadsura coccinea* (MH029822) was 145,413 bp in length. The overall GC content of the chloroplast DNA was 39.7%. The detected sequence length difference is predominantly attributable to the variation in the length of the intergenic spacer regions (Li and Zheng 2018).

In order to confirm the phylogenetic position of *Kadsura coccinea*, a maximum likelihood analysis was performed by MEGA 6.0 (Tamura et al. 2013) with 1000 bootstrap replicates (Minh et al. 2013; Chernomor et al. 2016) based on 13 complete chloroplast genomes. All sequences were aligned with the HomBlock pipeline (Bi et al. 2018) and subsequently checked manually in Bioedit v5.0.9 (Hall 1999). The results showed that *Kadsura coccinea* was sister to *Kadsura longipedunculata* with 100% bootstrap support (Figure 1).

**Figure 1.**
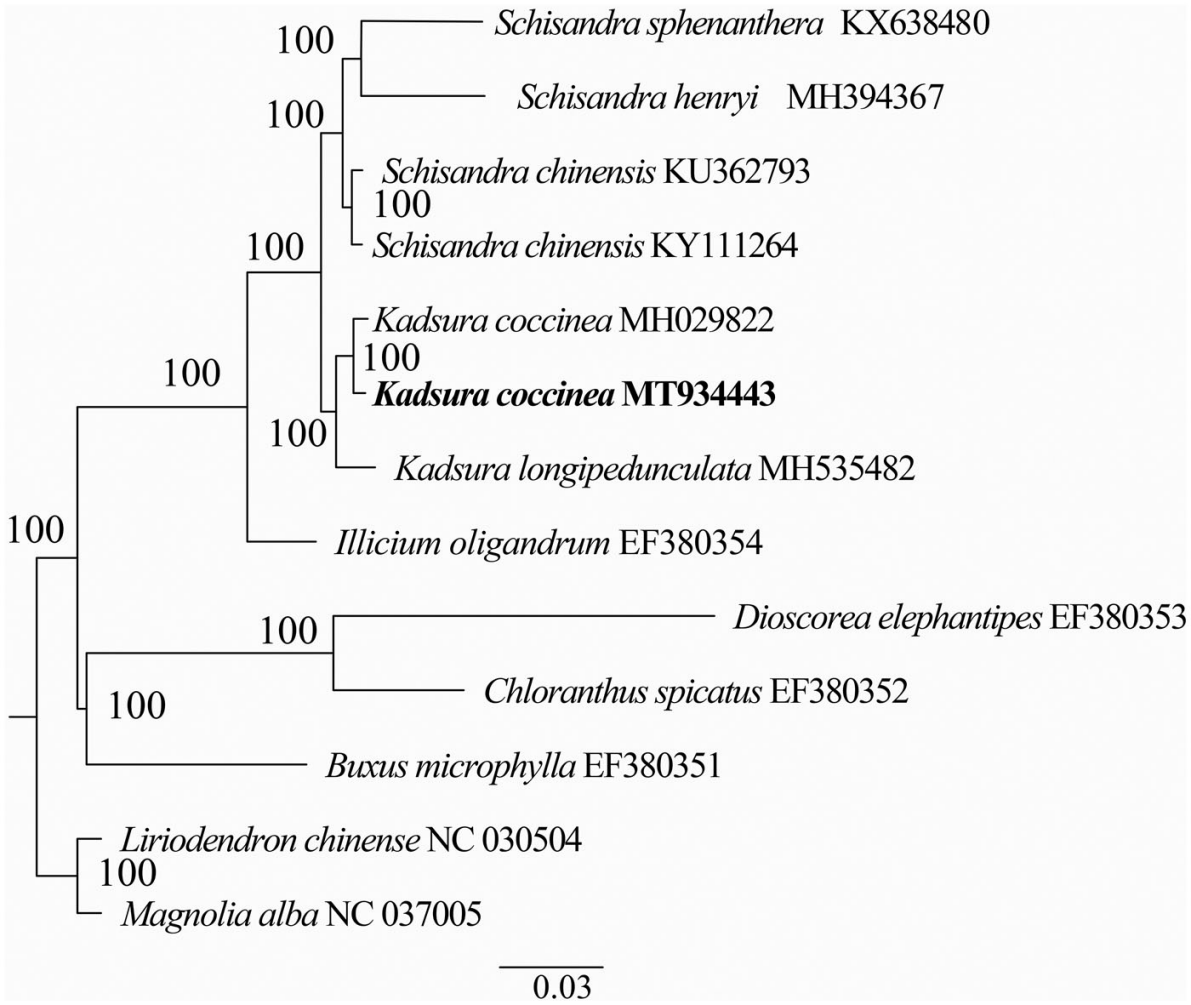
A Phylogenetic tree was constructed based on 13 complete chloroplast genome sequences. All the sequences were downloaded from NCBI GenBank.

## Data Availability

Data openly available in a public repository that does not issue DOIs. The data that support the findings of this study are openly available in [National Center for Biotechnology Information] at [https://www.ncbi.nlm.nih.gov/], reference number [MT934443]. BioSample metadata are available in the NCBI BioSample database (http://www.ncbi.nlm.nih.gov/biosample/) under accession number SRR13766590.
